# Malaria infection promotes a selective expression of kinin receptors in murine liver

**DOI:** 10.1186/s12936-019-2846-3

**Published:** 2019-06-24

**Authors:** Priscilla D. S. Ventura, Carolina P. F. Carvalho, Nilana M. T. Barros, Leonardo Martins-Silva, Edilson O. Dantas, Carolina Martinez, Pollyana M. S. Melo, João B. Pesquero, Adriana K. Carmona, Marcia R. Nagaoka, Marcos L. Gazarini

**Affiliations:** 1 Departamento de Biociências, Universidade Federal de São Paulo, Rua Silva Jardim 136, Lab 329, 3ºandar, Vila Mathias, Santos, 11015020 Brazil; 2 Departamento de Ciências Biológicas, Universidade Federal de São Paulo, Diadema, Brazil; 3 Departamento de Medicina, Universidade Federal de São Paulo, São Paulo, Brazil; 4 Departamento de Biofísica, Universidade Federal de São Paulo, São Paulo, Brazil

**Keywords:** *Plasmodium*, Malaria, Kinin receptors, Liver, Captopril

## Abstract

**Background:**

Malaria represents a worldwide medical emergency affecting mainly poor areas. *Plasmodium* parasites during blood stages can release kinins to the extracellular space after internalization of host kininogen inside erythrocytes and these released peptides could represent an important mechanism in liver pathophysiology by activation of calcium signaling pathway in endothelial cells of vertebrate host. Receptors (B1 and B2) activated by kinins peptides are important elements for the control of haemodynamics in liver and its physiology. The aim of this study was to identify changes in the liver host responses (i.e. kinin receptors expression and localization) and the effect of ACE inhibition during malaria infection using a murine model.

**Methods:**

Balb/C mice infected by *Plasmodium chabaudi* were treated with captopril, an angiotensin I-converting enzyme (ACE) inhibitor, used alone or in association with the anti-malarial chloroquine in order to study the effect of ACE inhibition on mice survival and the activation of liver responses involving B1R and B2R signaling pathways. The kinin receptors (B1R and B2R) expression and localization was analysed in liver by western blotting and immunolocalization in different conditions.

**Results:**

It was verified that captopril treatment caused host death during the peak of malaria infection (parasitaemia about 45%). B1R expression was stimulated in endothelial cells of sinusoids and other blood vessels of mice liver infected by *P. chabaudi*. At the same time, it was also demonstrated that B1R knockout mice infected presented a significant reduction of survival. However, the infection did not alter the B2R levels and localization in liver blood vessels.

**Conclusions:**

Thus, it was observed through in vivo studies that the vasodilation induced by plasma ACE inhibition increases mice mortality during *P. chabaudi* infection. Besides, it was also seen that the anti-malarial chloroquine causes changes in B1R expression in liver, even after days of parasite clearance. The differential expression of B1R and B2R in liver during malaria infection may have an important role in the disease pathophysiology and represents an issue for clinical treatments.

## Background

Malaria is an important health issue causing a considerable burden worldwide. The symptoms of the disease are related to the erythrocytic stages of *Plasmodium*, which comprises host cell invasion, haemoglobin consumption, parasite multiplication and erythrocytes rupture, resulting in an anaemic fever with intricate signaling pathways [1, 2]. After *Plasmodium* invasion, erythrocytes undergo cell modifications, such as formation of trafficking routes (tubovesicular network) for molecules [3, 4], modulation of ion homeostasis [5, 6] and addition of variant proteins in the host cell membrane [3, 7, 8].

Some host pathophysiological responses observed during malaria disease comprises coagulopathy, accumulation of leukocytes in the cerebral microcirculation, blood–brain barrier leakage, cerebral vasoconstriction and edema [9–11]. The inflammatory response to *Plasmodium falciparum* is also associated with red blood cells (RBCs) rupture, and release of parasite metabolites in the plasma, inducing a systemic inflammatory response [12, 13].

During the liver stage of malaria infection, the sporozoite parasites released from female *Anopheles* mosquitoes bite, reach the hepatocytes for infection, differentiation and multiplication of merozoites, which are released by hepatocyte rupture in the hepatic sinusoids for further erythrocytic invasion [14]. Malaria infection triggers hypertrophy of Kupffer cells, swelling of hepatocytes, sinusoidal cell necrosis and loss of hepatocyte microvilli [15, 16]. Kim et al. using a murine malaria model to investigate pathological mechanisms of liver injury showed the elevation of fibrogenesis related to hepatic stellate cells signalling activation [17].

During malaria development, the parasite metabolism produces haemozoin and haem, as major metabolites released from each parasite egress from erythrocyte, which in turn resulted from large haemoglobin degradation in parasite food vacuole [18, 19]. These molecules induce a transcription of inflammation-inducible genes in liver and they are a source of oxidative damage of liver cells [18, 20, 21]. The large amount of haemozoin presence in infected mice leads to hyperplasia of liver Kupffer cells [18]. Haemozoin can be maintained in liver and in other organs during months (at least 6), even after parasite clearance with chloroquine treatment [22, 23]. Haemozoin can also activates monocytes, neutrophils, dendritic cells and endothelial cells to secrete pro-inflammatory cytokines (TNF, IFN-γ, IL-1β), and recruiting CD4 and CD8 T cells [13, 24, 25].

At the beginning, this inflammatory response is beneficial, reducing the parasite growth and activating catabolic pathways to eliminate parasite toxins and host molecules, which may be dangerous in large quantities [13, 20]. The increased number of infected erythrocytes interferes in haemodynamics, by establishing the anaemia state, the erythrocyte sequestration in microvasculature and the activation of endothelial cells [26, 27]. These events related to haemodynamics changes are still poorly understood and studied in malaria pathogenesis.

The hepatic microcirculation can be modulated by the kinin-signalling pathway through activation of B1 or B2 kinin receptors resulting in a portal hypertensive response [28]. Both receptors are coupled to G protein, but the B2R is constitutively expressed, whereas the B1R expression is inducible by inflammatory cytokines in different tissues such as smooth muscle cells, endothelial cells, human sinusoidal cells from fibrotic liver and others [27, 29, 30].

Information about kinin involvement in malaria infection is scarce. It was shown previously that *Plasmodium* can internalize and hydrolyze host plasma kininogen releasing Lys-BK, des-Arg^9^-BK and BK through the parasite cysteine proteases, falcipain-2 and falcipain-3 [31]. On the other hand, captopril, the specific angiotensin I-converting enzyme (ACE) inhibitor, leads to an increase in BK levels in cell culture and compromises the erythrocyte invasion by *P. falciparum* [32]. A correlated study shows that BK and derivate peptides affect membrane integrity of *Plasmodium gallinaceum* sporozoites [33]. In addition, the invasion of erythrocytes by *P. falciparum* in culture can be impaired in a dose-dependent manner by angiotensin II, Ang 1–7 and BK peptides [34].

In this context, the aim of this study was to investigate the expression and immunolocalization of B1R and B2R receptors profiles in the host liver during *Plasmodium chabaudi* infection and the effect of the ACE inhibition by captopril in association or not with the anti-malarial chloroquine on mice survival. This study can elucidate whether occurs a selective modulation of liver subareas by infected erythrocyte, increasing the understanding of hepatic response and modifications during the malaria infection.

## Methods

### Reagents

Captopril and chloroquine were purchased from Sigma Chemical Co (UK). The ACE fluorogenic substrate Abz-FRK(Dnp)P-OH from AminoTech (São Paulo, Brazil).

### Animals

Adult male Balb/c mice weighing 25–30 g were maintained at controlled temperature (22 ± 2 °C), 60–90% humidity under a 12 h light/dark cycle and received feed and water ad libitum. The mice were infected by *Plasmodium chabaudi*, and the control group (CTL) corresponds to uninfected mice. Chloroquine (CQ) and captopril (CP) were tested alone or in association in infected mice by daily oral gavage for 15 days. The experimental design is composed by the following groups: CTL (uninfected mice); INF (infected mice treated with saline solution/0.9% NaCl), CP (infected mice treated with captopril 45 mg/kg/day), CQ (infected mice treated with chloroquine 20 mg/kg/day) and CQ + CP (infected mice treated with captopril and chloroquine at the same previous concentrations).

### Kinin B1 and B2 receptor knockout mice survival during malaria infection

To further investigate the importance of B1R and B2R during malaria infection B1R and B2R knockouts [35, 36] and wild-type mice was also studied. Male C57BL/6 mice age 3 months from CEDEME–UNIFESP maintained on a light–dark 12:12 cycle under controlled temperature (20 ± 2 °C) with free access to food and water. Survival curves for knockout B1R, B2R and wild-type mice were performed with infection (i.p. 10^3^ cells in PBS solution) with *P. chabaudi* (n = 8 animals per group). For cumulative survival experiments, the statistical significance among wild type and B1R and B2R knockouts was analysed with Log-Rank test using Prism™ version 4.03 for windows (GraphPad, USA).

### *Plasmodium chabaudi* maintenance and isolation

*Plasmodium chabaudi* (clone AJ) was maintained in Balb/C mice by weekly transfer of infected blood to a healthy mice. Animals infected at the trophozoite stage (parasitaemia ~ 60%) were euthanized and leukocytes and platelets were removed from whole blood by filtration through a powdered cellulose column (Whatman CF11). Trophozoite-infected erythrocytes were then washed three times by centrifugation at 1500*g* for 5 min in phosphate buffered saline (PBS; 137 mM NaCl, 2.7 mM KCl, 4.3 mM Na_2_HPO_3_, 1.4 mM Na_2_HPO_3_, pH 7.4). Parasites (10^7^ cells/ml) were isolated by erythrocyte membrane lysis with 10 mg/ml saponin in PBS for 10 min. After pelleting to remove red cell membranes, the parasites were washed twice in PBS by centrifugation at 2000*g* at 4 °C for 5 min.

### Plasma ACE proteolytic activity

Blood of each group of animals (control and treated groups) was collected at the infection peak (about 9th day) in tubes with ACD solution (60 mM sodium citrate; 30 mM citric acid; 60 mM d-glucose) for plasma separation with 20 min centrifugation at 4 °C and 1500 rpm. The ACE activity measurement was performed as previously described [37]. Plasma (5 µl) were added to 0.1 M Tris–HCl, containing 50 mM NaCl and 10 µM ZnCl_2_, pH 7.0, in a final volume of 1.0 ml. The reaction was initiated by the addition of 10 µM of the substrate Abz-FRK(Dnp)P-OH, and the enzyme hydrolytic activity was continuously monitored in a Hitachi F-7000 spectrofluorimeter (λ_ex_ = 320 nm and λ_em_ = 420 nm). The rate of substrate hydrolysis was measured as Arbitrary Fluorescence Units (AFU/min) in a 96 well microplate.

### Immunolocalization of B1 and B2 receptors in liver

Liver fragments were frozen in *n*-hexane cooled with dry ice and were stored at − 80 °C until cryotomy. For B1R and B2R immunolabelling, the liver sections (8 µm thickness) were fixed with acetone at − 20 °C for 3 min followed by washing with TBS and permeabilization with 0.1% Triton X-100 in TBS (Tris buffered saline containing 0.05 M Tris–HCl and 150 mM NaCl, pH 7.4) for 5 min. All sections were then incubated for 1 h with 5% BSA in TBS 0.05 M, pH 7.4 prior to incubation overnight at room temperature (RT) with one of the following primary antibodies: goat anti-B1R and rabbit anti-B2R (Santa Cruz Biotechnology, USA) diluted 1:30 in 3% BSA in TBS. For B1R staining the sections were incubated for 2 h at RT with a mouse anti-goat/sheep (1:150) followed by anti-mouse FITC-conjugated tertiary antibody incubation (1:50; 2 h at RT). For B2R, slices were incubated at RT (2 h) with anti rabbit FITC-conjugated secondary antibody (Sigma Chemical Co, USA; dilution 1:150).

All sections were mounted with antifading (Vectashield, Vector Laboratories, Burlingame, USA) and observed in a microscope Axio Observer (Carl Zeiss, Germany) with MRc colour camera. For negative control (CNeg), liver sections of control mice (CTL—uninfected group) were incubated only with secondary antibody (for B2R reaction) and with secondary and tertiary antibodies for B1R reaction. The semiquantitative analysis of the immunohistochemistry data was analysed with at least 6 images from 3 to 4 independent experiments (immunohistochemistry assays), using liver sections of 3–4 mice from group. In each picture were analysed the integrated density of fluorescence of 60 points placed at the B1R or B2R staining exhibited by vessels or sinusoids.

### Western blot for B1 and B2 kinin receptors

Liver fragments were homogenized in the lysis buffer containing 50 mM Tris–HCl, 150 mM NaCl, 1% Nonidet P-40, 0.1% SDS, 0.5% deoxycholic acid and protease inhibitor cocktail (Sigma Chemical Co, USA). The homogenized tissue was centrifuged at 14,000 rpm and 4 °C. The protein content was measured using BCA protein assay (Pierce Protein Biology Products). Total proteins (30 µg for B1R and 50 µg for B2R) were separated by 10% SDS-PAGE and transferred to a nitrocellulose membrane (Bio-Rad, USA). After washing with TTBS (0.05 M Tris–HCl, 150 mM NaCl, 0.1% Tween 20, pH 7.4), the membranes were blocked for 4 h at room temperature (RT) with 5% BSA in TTBS and incubated overnight with polyclonal goat anti-B1R (1:1000 dilution) or polyclonal rabbit anti-B2R (1:1000) (Santa Cruz Biotechnologies) in 3% BSA/TTBS solution at RT. After, the membranes were incubated with secondary antibodies conjugated to horseradish peroxidase diluted 1:5000 (anti-goat) and 1:2500 (anti-rabbit) for 2 h at RT. The peroxidase reaction was detected with ECL substrates (Biorad, USA) using a chemiluminescence documentation system (Uvitec Limited, Cambridge, UK). The immunoreactive bands were analysed by ImageJ software (National Institute of Health), and the densitometric results normalized with the respective internal control signals (GAPDH, Sigma, USA). The quantification of B1R expression was performed using 4 membranes with samples from 3 mice per group and B2R was analysed with 5 membranes from samples of 4 mice per group.

### Statistical analysis

Results are expressed as mean ± SEM. Data were analysed using one-way ANOVA followed by the Bonferroni’s post-test (Graph Prism software version 6.0) with parametric data. To analyse nonparametric data Kruskal–Wallis was used followed by Dunn’s multiple comparison test. Significance was indicated when value of p ≤ 0.05.

## Results

### Effect of oral administration of captopril in combination with chloroquine on *Plasmodium chabaudi* AJ mice infection

**Fig. 1:**
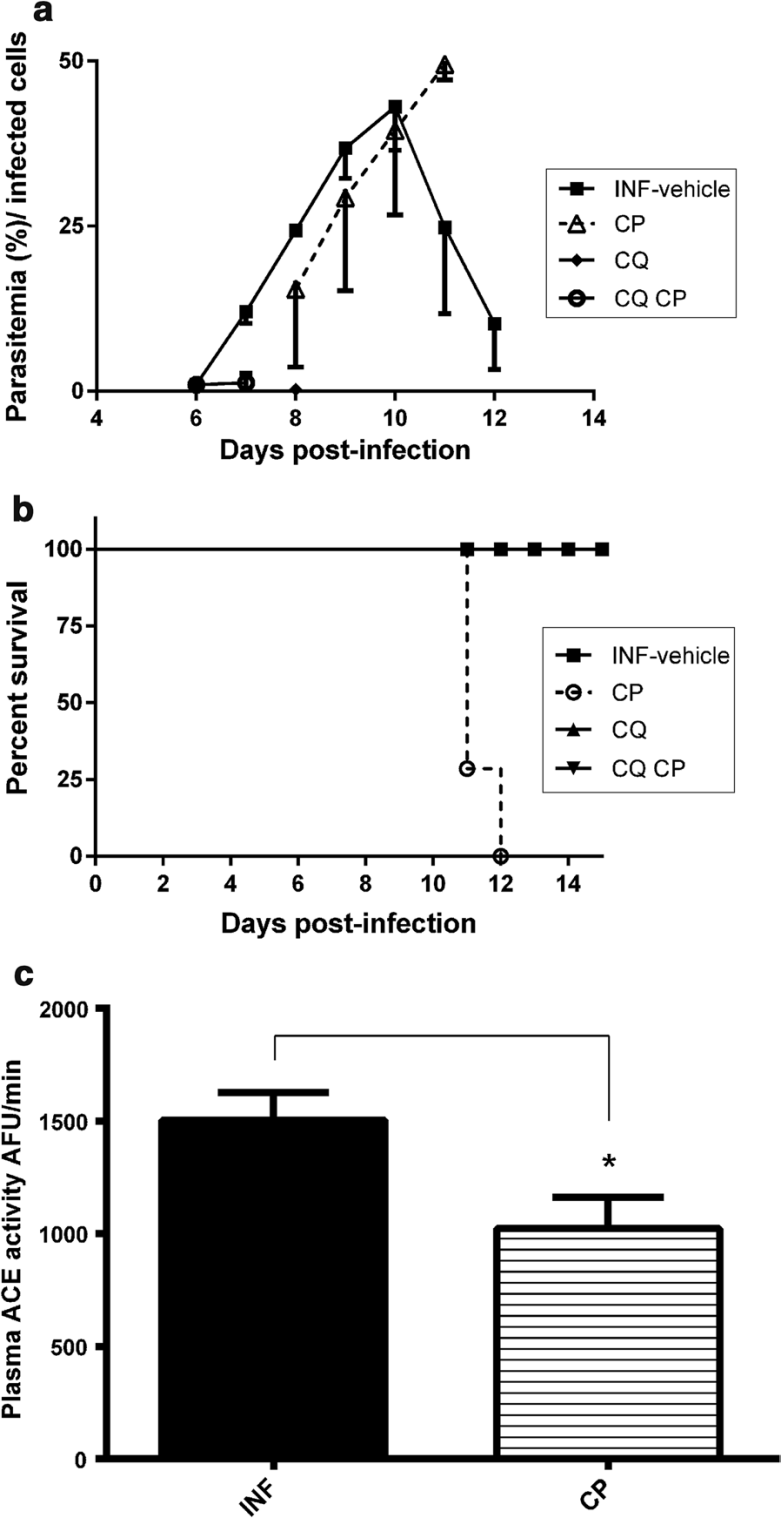
*Plasmodium chabaudi* parasitaemia in mice treated or not with captopril, chloroquine or both drugs (n = 6). **a** Blood parasitaemia of infected groups with vehicle solution (INF), captopril (CP), chloroquine (CQ), chloroquine and captopril association (CQ CP) (n = 6). **b** Survival curve of each animal group showed in A (n = 6). **c** ACE activity measured with Abz-FRK(Dnp)P-OH in blood plasma from mice of infected groups with vehicle solution (INF) or treated with CP. (n = 3). One-way ANOVA with multiple comparison post-test of Bonferroni’s; *p < 0.05, compared with the respective control of treatment (INF, without CP)

The oral treatment was initiated after the confirmation of infected erythrocytes in the blood smear stained with Giemsa. In Fig. 1a, the infected (INF) group showed highest parasitaemia (45%) between the 9th and 11th day, followed by a progressive regression and total recovery, indicating that the immune system was able to control and cure the infection. Chloroquine (CQ) and chloroquine + captopril (CQ + CP) groups showed low parasitaemia in the initial days and clearance of *P. chabaudi* after 3 days of infection. However, parasitaemia of the CP group reached 45% at 11–12th day of infection. The survival rate (%) of animals is shown in Fig. 1b, and it is possible to verify that the treatment with CP (45 mg/kg/day) resulted in the death of the animals (100%) during the parasitaemia peak (about 45%), while groups with CQ treatment survived.

To confirm the ACE inhibition the blood plasma samples from each group, INF (infected + vehicle) and CP (infected mice treated with 45 mg/kg captopril) were obtained at the 11th day (infection peak) and tested with the ACE substrate Abz-FRK(Dnp)P-OH (Fig. 1c). The oral administration of captopril decreased significantly (35%) the plasma ACE activity in infected mice when compared with the control group (Fig. 1c).

### Survival of B1R and B2R knockout mice infected by *P. chabaudi*

**Fig. 2:**
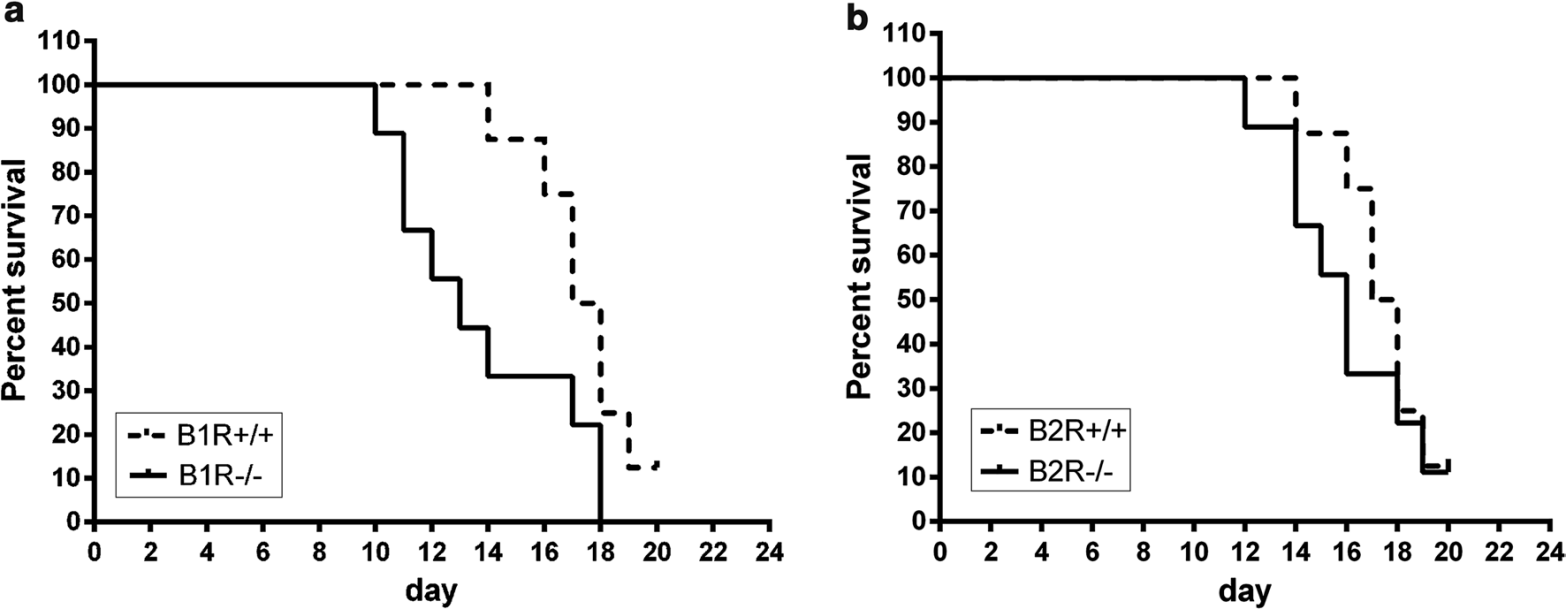
Survival of bradykinin receptor B1 and B2 knockout mice infected by *Plasmodium chabaudi*. Each group (knockout and wild type) consisted of C57BL6 (n = 8) infected i.p. with 10^3^ iRBC. **a** Survival curve of B1 receptor knockout mice *vs* wild type infected mice (*p *< 0.05). **b** Survival curve of B2 receptor knockout mice *vs* wild type infected mice (*p *> 0.05; ns). The statistical significance of difference between control and experimental groups was determined with the Log-Rank test for the cumulative survival experiments

In order to address the relevance of kinin receptors signalling pathways in the survival rate B1R and B2R knockout mice were followed during *P. chabaudi* infection. The results indicated that B1R knockout mice (B1R−/−) presented a significant reduction of survival when compared to its respective control (wild type mice). Differently, no significant difference was verified between B2R knockout mice (B2R−/−) and wild type mice Fig. 2.

### Immunolocalization and quantification of B2 kinin receptors in liver of infected and uninfected mice

**Fig. 3:**
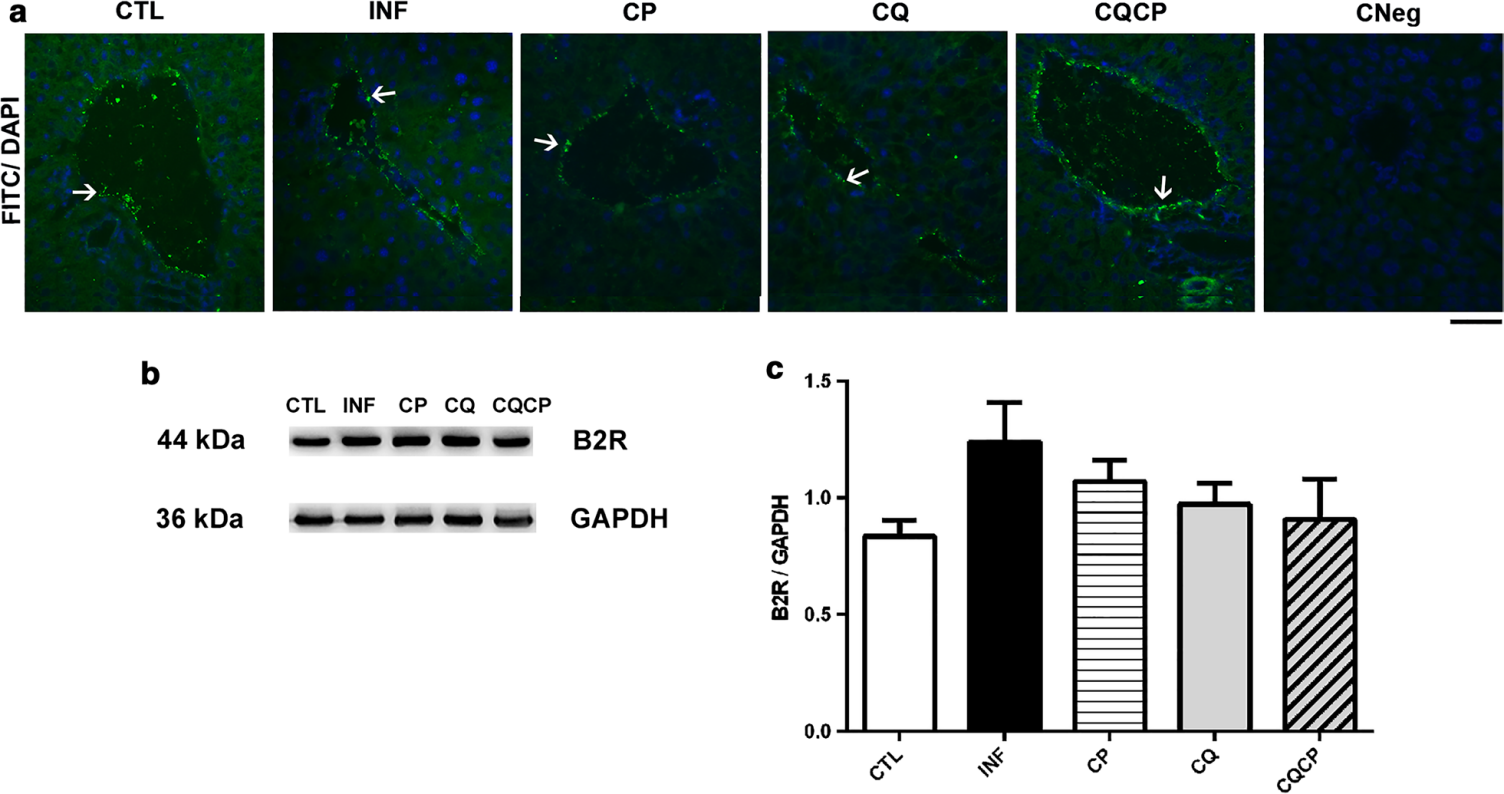
B2 kinin receptor expression in liver of uninfected and infected mice by *Plasmodium chabaudi*. **a** Cellular location of B2 kinin receptor in liver of uninfected group (CLT) and infected-treated with 0.9% NaCl (INF), captopril (CP), chloroquine (CQ) or chloroquine and captopril association (CQCP). Negative control (CNeg) was performed in liver of control mice. The white arrows indicate B2R staining in vessel walls (green). **b**, **c** Expression of B2R determined in liver homogenate by western blotting and respective densitometric quantification of immunoreactive bands for B2R immunoblottings (**c**). All values were normalized by GAPDH bands (36 kDa, internal control). n = 5 for B2R mean ± SEM

The B2R immunolocalization and expression in liver of infected and uninfected mice treated with captopril, chloroquine or both drugs in association was analysed (Fig. 3). Figure 3a shows the immunolocalization of the constitutive expressed B2 receptor (B2R) in blood vessels of all groups, it is not possible to confirm the presence of B2R in sinusoid walls, but neither state its absence. The B2R staining detected in sinusoidal spaces is low, making it difficult to individualize the sinusoidal spaces. The control group without the primary antibody (anti-B2R) did not present any fluorescence (CNeg). Figure 3c did not demonstrate statistical differences concerning liver B2R expression among the experimental groups studied.

### Immunolocalization and quantification of B1 receptors in liver of infected mice and uninfected mice

**Fig. 4:**
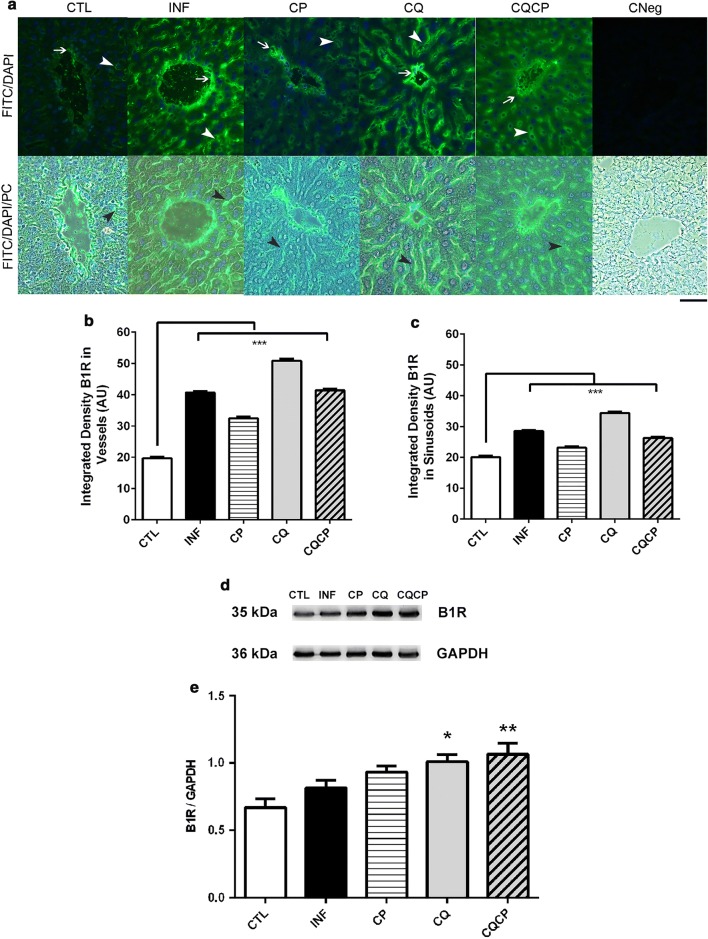
B1 kinin receptor expression in liver of mice infected with *Plasmodium chabaudi*. **a** Immunofluorescence of B1 receptor (green) in liver tissue. The white arrows indicate the B1R immunostaining in blood vessels, and the arrowheads (black or white) show the sinusoidal spaces. Semi-quantitative analysis of B1R density in vessels (**b**) and sinusoids (**c**). DNA fluorescence (DAPI/blue) and phase contrast (PC). n = 3/4 mice per group. Kruskal–Wallis with multiple comparison post-test of Dunn’s; ***p < 0.0001. **d**, **e** Densitometric quantification of bands for B1R. The values represent mean ± SEM (n = 4); One-way ANOVA test with post-test of Bonferroni’s *p < 0.05 and **p < 0.001 compared with CTL

Immunolocalization of kinin B1 receptor in mice liver was performed for control group (uninfected mice) and also for infected groups submitted or not (INF + vehicle) to treatments with chloroquine (CQ), captopril (CP) or chloroquine + captopril (CQCP) (Fig. 4a). It was detected a strong fluorescence signal for B1R in sinusoids (arrowheads) and other blood vessels walls (arrows) of the infected groups (treated with drugs or not). Importantly, the CTL group showed a very low intensity for B1R in both regions, indicating that *Plasmodium* infection modulated the B1R content in liver. The representative photomicrographs of CNeg (without primary antibody incubation) showed the negative control of the reaction in liver of CTL mice. The blood vessel walls with luminal linear length up to 300 µm was analysed (fluorescence quantification) (Fig. 4b, c). The quantifications (in sinusoids or other blood vessels) confirm the differences between the control and treated groups. Interestingly, it was observed an increased signal for B1R in liver of mice treated with CQ, when compared with other groups, even after total clearance of blood parasitaemia (Fig. 4d, e).

## Discussion

The investigations on malaria infection carried out in the recent years aimed for a better understanding of the complex relationship between *Plasmodium* and host. The ability of *P. falciparum* to internalize human plasma kininogen followed by the release of active kinins was reported [31]. In the present study, it was demonstrated the effect of treatment with the ACE inhibitor captopril, associated or not with chloroquine (an anti-malarial drug) during malaria infection and its role on kinin system in the liver.

The survival results demonstrated that mice were unable to recover from malaria infection when treated with captopril. The use of this antihypertensive drug leads to mice death at the parasitaemia peak, around the 11th day. The effect of captopril has already been studied during malaria infection on pro-inflammatory cytokines production and did not demonstrate a significant effect [32].

The hepatic degradation of BK occurs mainly by ACE and promotes a reduction of BK hypertensive action in sinusoidal endothelial cells [38]. Thus, ACE inhibitors have been used as essential therapeutic agents for cases of systemic hypertension [39]. The cardiovascular protective action of these compounds is due to the inhibition of Ang II formation regulated by local and systemic RAS (renin–angiotensin-system). Inhibition of Ang II was related to the endothelial function improvement in different experimental models [40, 41].

Despite these previous findings of RAS signaling pathways in vertebrate models, in malaria disease these signalling molecules such as Ang II promotes a reduction of erythrocyte invasion by *Plasmodium falciparum* in culture and are related to an increase of Ang (1–7) and BK in the culture supernatant of *Plasmodium falciparum* after addition of captopril [32–34].

Kinin B1R is an important inflammatory mediator and is correlated to the hemodynamic response to kinins, with increased blood pressure induced by des-Arg^9^-bradykinin [42, 43]. In hepatic perfusion, BK induces a portal hypertensive response that is mediated by B2R and modulated by NO system in healthy liver, as well as, in inflammatory, fibrotic, and cirrhotic conditions [28]. The B1R and B2R antagonists promote downregulation of pro-inflammatory molecules and upregulation of anti-inflammatory molecules, representing a new therapeutic strategy for the prevention and treatment of renal ischemia-warm reperfusion injury [44]. It has also been described the antifibrogenic effect of BK on the experimental model of chronic liver injury [30]. Interestingly, the parasites can process kininogen and release des-Arg^9^-bradykinin and bradykinin [31], which could also contribute to attenuate the liver damage [30].

The absence of inducible B1R reduces the mice survival during *P. chabaudi* infection (Fig. 2), which is probably related to the endothelial vascular dysfunction associated to changes in NO production and excessive ROS generation [45]. In inflammation models, B1R are involved in cell protection effects [30, 36, 46].

**Fig. 5:**
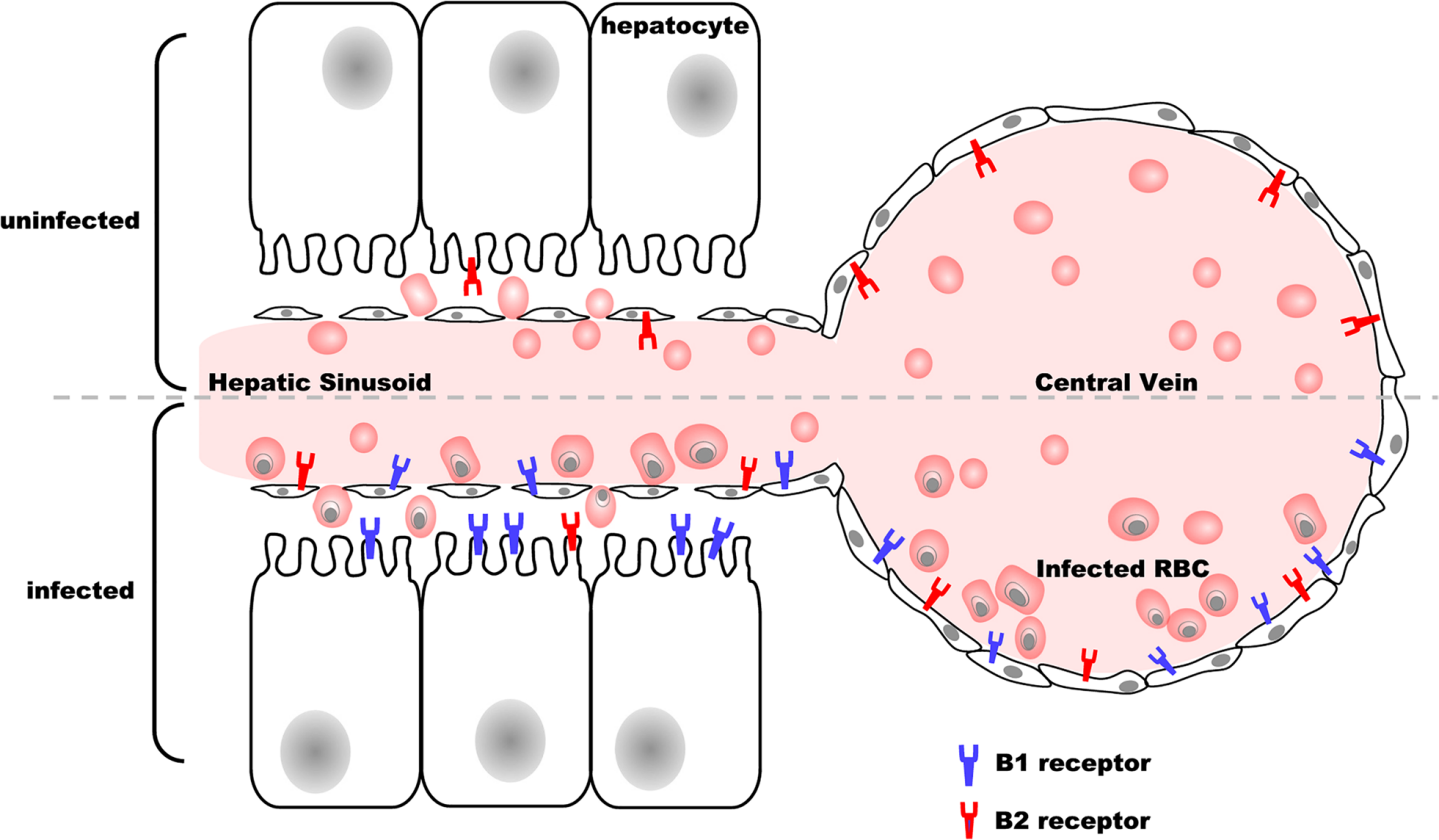
Schematic model of kinin receptors distribution in the liver cells during murine malaria infection. Immunolocalization B1 and B2 receptors in mouse liver uninfected and infected with *P. chabaudi* from the results obtained at peak infection

The selective localization pattern of B1 receptors (in vessels and sinusoids) and B2R (only in vessels) in mice infected by *P. chabaudi* indicate some important alterations, which occur during malaria infection. The cell signaling resulting from this differential expression of kinin receptors is still under investigation, but could be related to the prolonged inflammatory state associated with high haemozoin concentration maintained in many organs for at least 6 months [22]. Haemozoin released from hemoglobin metabolism by parasites [19, 23] can induce the production of pro-inflammatory mediators, such as TNF, IL-1 IFN-γ, IL-1β [24], which are involved in kinin receptor upregulation [47]. Figure 5 summarizes the main results showing the differential modulation of liver kinin receptors during malaria infection. These findings are relevant to further understanding of hepatic physiological responses during and after infection.

Another interesting finding is that mice treated with chloroquine presented an upregulation of B1R expression in liver, even after 8 days of parasite clearance, indicating that physiological changes in host tissue could be persistent for several days. The chloroquine action mechanism is not completely elucidated, but some studies show the CQ efficacy in the treatment of inflammatory diseases is due to the inhibition of pro-inflammatory cytokine production [48, 49]. However, if used in liver ischemia/reperfusion injury, chloroquine possesses a dual effect, presenting a protective role if administrated at an early phase, by reducing cytokine production and liver damage, but inducing apoptosis and inhibiting autophagy at late phase [50].

In erythrocytic cycle, the parasite proteins are exported to the surface of infected erythrocytes, providing the immune cell recognition, and also cell adhesion to the host vascular endothelium [51, 52]. This parasite-host interaction is associated with the pathology of malaria and contributes to the development of severe malaria [23]. Increased number of infected erythrocytes promotes the secretion of pro-inflammatory molecules and cytokines, and also induces the expression of adhesion molecules, such as E-selectin, ICAM-1 and V-CAM1 on the surface of endothelial cells [3].

The endothelial adhesion of infected erythrocytes in the microvasculature of different organs contributes to the parasite evade of spleen clearance [53]. Animals infected with *Plasmodium berghei* ANKA, a cerebral malaria model, show a five-fold increase in plasma TNF concentration and an increase of ten times in IFN-γ levels [54]. Hence, the same study demonstrated that infected groups treated with losartan (antagonist of AT1 receptor) showed a partial suppression or blockade of IFN-γ and TNF production [54].

The vasodilatation caused by ACE inhibition can induces less infected RBC adhesion and diminished vessel obstruction by rosettes [55, 56]. However, the ACE inhibition has disadvantage roles such as, allowing fast intense inflammatory response, compromised oxygen transport to tissues, leading to hypoxia and an impaired cell metabolism in a situation of severe anaemia [55, 56]. Additionally, this study reports for the first time that administration of antihypertensive drugs should be managed with care to malaria patients.

## Conclusions

The reduced survival observed for mice treated with ACE inhibitor and by B1R knockout mice demonstrate the importance of haemodynamics modulation for the pathophysiology of the disease. The selective distribution patterns of kinin receptors in liver also indicate one of the cell mechanisms involved during the infection response. Additionally, this study highlights some concerns about clinical treatment of malaria patients, which includes the attention for hepatic pathophysiological changes even after blood parasite clearance, and also for the usage of hypotensive drugs during *Plasmodium* infection.

## Data Availability

Not applicable.

## Abbreviations

RBCs: red blood cells
iRBC: infected red blood cells
ACE: angiotensin I-converting enzyme
B1R: B1 kinin receptor
B2R: B2 kinin receptor
BK: bradykinin
PKA: protein kinase A
AngII: angiotensin II
Ang (1–7): angiotensin 1–7
CQ: chloroquine
CP: captopril
GAPDH: glyceraldehyde-3-phosphate dehydrogenase
RT: room temperature
